# The Regulation of rRNA Gene Transcription during Directed Differentiation of Human Embryonic Stem Cells

**DOI:** 10.1371/journal.pone.0157276

**Published:** 2016-06-14

**Authors:** Jessica L. Woolnough, Blake L. Atwood, Zhong Liu, Rui Zhao, Keith E. Giles

**Affiliations:** UAB Stem Cell Institute, Department of Biochemistry and Molecular Genetics, The University of Alabama at Birmingham, Birmingham, Alabama 35294, United States of America; University of Louisville, UNITED STATES

## Abstract

It has become increasingly clear that proper cellular control of pluripotency and differentiation is related to the regulation of rRNA synthesis. To further our understanding of the role that the regulation of rRNA synthesis has in pluripotency we monitored rRNA synthesis during the directed differentiation of human embryonic stem cells (hESCs). We discovered that the rRNA synthesis rate is reduced ~50% within 6 hours of ACTIVIN A treatment. This precedes reductions in expression of specific stem cell markers and increases in expression of specific germ layer markers. The reduction in rRNA synthesis is concomitant with dissociation of the Pol I transcription factor, UBTF, from the rRNA gene promoter and precedes any increase to heterochromatin throughout the rRNA gene. To directly investigate the role of rRNA synthesis in pluripotency, hESCs were treated with the Pol I inhibitor, CX-5461. The direct reduction of rRNA synthesis by CX-5461 induces the expression of markers for all three germ layers, reduces the expression of pluripotency markers, and is overall similar to the ACTIVIN A induced changes. This work indicates that the dissociation of UBTF from the rRNA gene, and corresponding reduction in transcription, represent early regulatory events during the directed differentiation of pluripotent stem cells.

## Introduction

Human embryonic stem cells contain a specialized chromatin structure, which is in part responsible for their unique ability to differentiate into most adult cell types, the defining feature of pluripotency [1–3]. Specifically, the chromatin structure within pluripotent stem cells is more dynamically regulated [4, 5] and global transcription is greater [6], relative to more differentiated cell types. The pluripotent state is maintained by a pluripotency-promoting transcriptional network, which consists of transcription factors such as OCT4, SOX2, and NANOG (1). These transcription factors activate expression of pluripotency-promoting genes and repress expression of lineage-specific genes through interaction with sequence-specific DNA binding sites and transcriptional cofactors [1]. Pluripotent stem cells can be induced to adopt specific differentiation programs *in vitro* through either growth in the presence of extra-cellular signaling molecules or by the over-expression of lineage specific transcription factors [7–10].

The ribosome is composed of four non-coding RNAs, the 28S, 5.8S, 18S, and 5S rRNAs, which account for roughly 60% of the total RNA in any given cell [11]. The first three of these rRNAs are synthesized from a single 13 kb primary transcript, termed the 47S rRNA, which is transcribed from the rRNA gene. Human cells consist of roughly 200 haploid, head to tail, copies of the rRNA gene within the entire p-arm of chromosomes 13–15, 21 and 22. These genes are transcribed by RNA polymerase I (Pol I), which is recruited to the rRNA gene promoter by the combined action of the transcription factors SL1 (TIF-1B in mouse) and UBTF [12–15]. The recruitment of Pol I to the promoter ultimately requires RRN3/TIF-1A, which interacts with SL1 and Pol I, and is required for growth-factor-dependent control of rRNA synthesis [16–18]. In addition to regulating the recruitment of Pol I, UBTF can facilitate promoter escape [19], elongation rate [20], and can regulate the higher-order chromatin structure of the rRNA gene [21]. Electron micrographs initially illustrated the possibility that not all rRNA genes are bound by Pol I [22, 23], and psoralen cross-linking studies indicate that the rRNA genes exist in at least two distinct biochemical states [24, 25]. The inactive state is characterized by the binding of the heterochromatin-promoting complex, NoRC, [26] and CpG methylation [27, 28] of the rRNA gene promoter. In mouse, NoRC is recruited by a cis-acting promoter RNA (pRNA), which is roughly 200 nucleotides in length and consists of the transcribed rRNA gene promoter region [29]. The presence of NoRC at the promoter prevents binding of UBTF1 and SL1, and ultimately blocks Pol I binding. The function of the pRNA in human cells remains less well understood.

The overall rRNA synthesis rate is regulated during normal developmental processes [30]. One possible mechanism involves altering the ratio of active to silent copies of the rRNA gene [31–33], which are thought to be in the fully active state within pluripotent stem cells [32, 33]. Downregulation of both rRNA synthesis rate and active copy number are linked to the exit from pluripotency and differentiation [31, 32, 34–36]. Despite these observations the field lacks a mechanistic understanding of how hESCs initiate silencing of rRNA synthesis, how this silencing promotes the exit from pluripotency, and how it affects the expression of lineage-specific gene expression. In this study, we investigate the regulation of the rRNA genes in human embryonic stem cells (hESCs). We confirm that the exit from pluripotency and activation of endoderm-specific gene expression can be induced by treating H9 ESCs with the TGF-β family member, ACTIVIN A [37]. We report that ACTIVIN A treatment leads to a reduction of the rRNA synthesis rate within 6 hours, which precedes the reduction of specific pluripotency markers, and the increase in specific germ layer markers. Furthermore, ACTIVIN A treatment reduces the binding of UBTF to the rRNA gene promoter in hESCs prior to any increase in heterochromatin levels throughout the rRNA gene. Direct reduction of rRNA synthesis induces the expression of markers for all three germ layers, reduces the expression of pluripotency markers, and is overall similar to the ACTIVIN A induced changes.

## Results

### ACTIVIN A treatment of hESCs facilitates the exit from pluripotency and endodermal-specific gene expression

**Fig 1:**
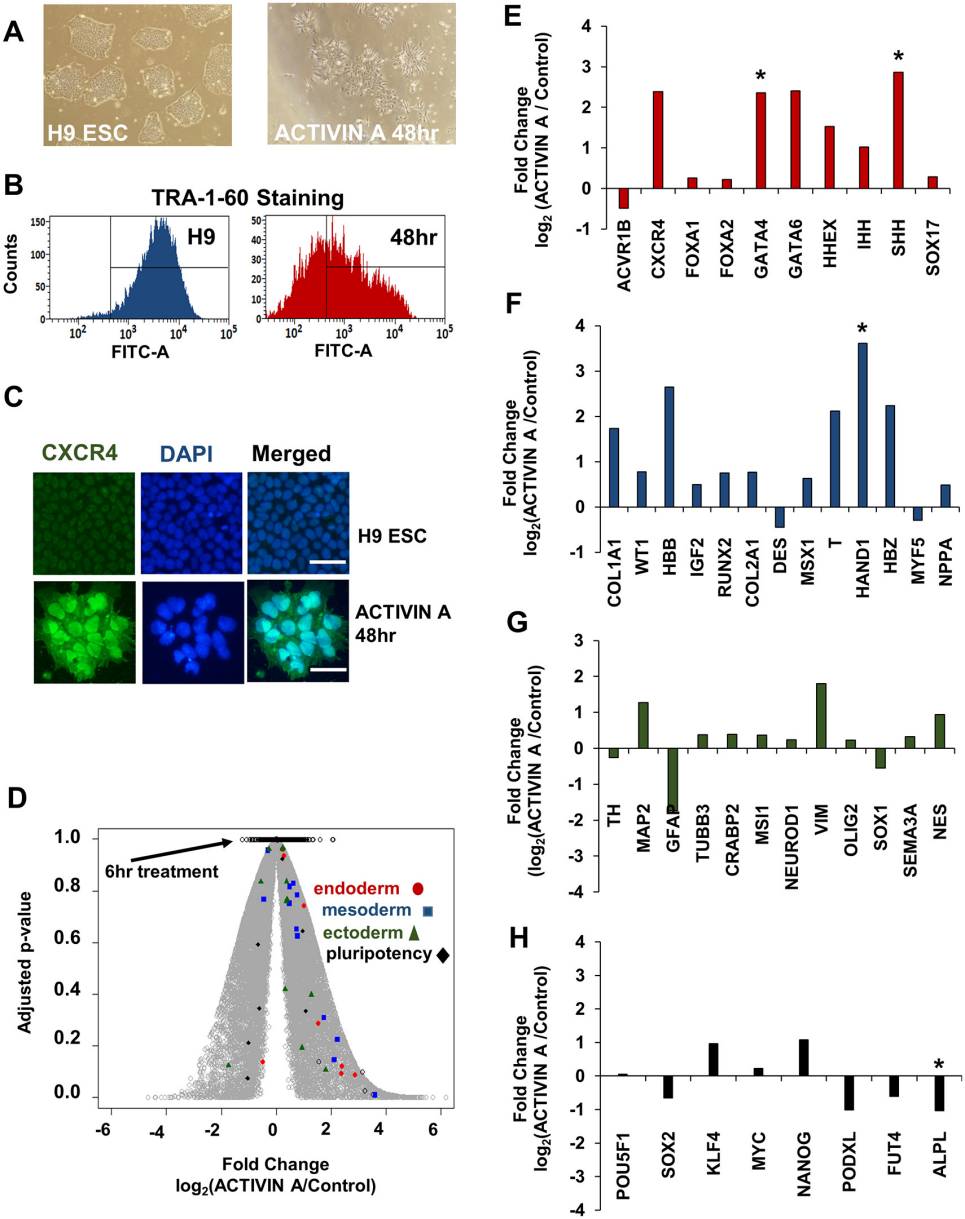
ACTIVIN A treatment of H9 ESCs induces endodermal specification and loss of pluripotency within 48 hours. (A) A phase-contrast image of untreated H9 ESCs (left) and 48 hour ACTIVIN A treated H9 ESCs (right) cultured as previously described [37] (See Methods). (B) FACS analysis of TRA-1-60 antigen (sc-21750, Santa Cruz Biotech) of the two cell types described in panel A using BD Fortessa Analyzer. The y-axis indicates the number of cells and the x-axis indicates the FITC signal (TRA-1-60, untreated H9 ESCs: 93.1% +/- 0.92 positive vs ACTIVIN A: 51.2% +/- 1.1 positive; N = 2, p = 0.0076). The quantitation was limited to live cells by first removing debris and dead cells. (C) Immunostaining of the endodermal marker CXCR4 on untreated and 48 hour ACTIVIN A treated H9 ESCs. Images were acquired using identical exposure conditions for untreated and treated cells. Scale bars, 100 μm. (D) A volcano plot is shown, which presents the significance of each genes change in expression (p-adjusted) as a function of its fold change. The clear circles with black outlines, mostly found at the top of the plot, represent the change in gene expression after 6 hours. The filled gray circles indicate the change in gene expression after 48 hours. Genes from endoderm (filled red circles), mesoderm (filled blue squares), ectoderm (filled green triangles), and pluripotency-markers (filled black diamonds) are indicated within the plot. (E-H) The fold change in expression after 48 hours of ACTIVIN A treatment is shown for endoderm (E), mesoderm (F), ectoderm (G), and pluripotency markers (H). Significance testing was performed within DESeq2 using the Benjamini/Hochberg correction to generate the adjusted p-value (p-adjusted), which represents a false discovery rate (FDR) of 10% [40].

To investigate the relationship between rRNA synthesis, the exit from pluripotency, and the induction of lineage-specific gene expression, we tested our ability to induce differentiation of hESCs towards the endodermal lineage using a previously published protocol of treating H1 hESCs with ACTIVIN A (50 ng/ml) [37]. We demonstrated that after 48 hours of ACTIVIN A treatment (Methods), H9 ESCs lost their ESC-specific colony morphology and developed a spindle-like appearance (Fig 1A). Consistent with their change in morphology, the H9 ESCs were 93.1% +/- 0.92 for TRA-1-60 staining, compared to 51.2 +/- 1.1 after 48 hours of ACTIVIN A treatment (Fig 1B, Student’s t-test, N = 2, p = 0.00076, only live cells were quantified). To confirm that the ACTIVIN A treatment of H9 ESCs was inducing differentiation we tested for the expression of the endoderm-specific marker, CXCR4 [8, 38]. We were unable to detect surface expression of CXCR4 within 48 hours, which is consistent with previous descriptions of the kinetics of endodermal differentiation (data not shown, [39]). However, indirect immunofluorescence clearly demonstrated that CXCR4 levels were increased intracellularly within 48 hours (Fig 1C).

To generate a more precise understanding of the timing of transcriptional changes induced upon ACTIVIN A treatment, we performed polyA+ RNA-seq on 0 hour-, 6 hour-, and 48 hour- ACTIVIN A treated H9 ESCs. We used the DESeq2 algorithm to identify statistically significant changes to gene expression [40]. To increase our statistical power and ensure biological significance we included publically available RNA-seq data from untreated and 48 hour- ACTIVIN A treated H1 ESCs (2 replicates each, [37], GSE41009). A volcano plot, which presents the fold change as a function of the adjusted p-value for each gene (Methods, [40]), indicates that there is very little change in gene expression after 6 hours of ACTIVIN A treatment (Fig 1D). However, the number of significantly changed genes increased greatly by 48 hours. After 6 hours of ACTIVIN A treatment there were three significantly upregulated, and zero significantly downregulated genes: LEFTY1, LEFTY2, and RPA-559A3.6 (Fig 1D, S1 Supporting Information). The number of significantly upregulated and downregulated genes increased to 1097 and 712, respectively, after 48 hours ACTIVIN A treatment in H9 ESCs (Fig 1D, S1 and S2 Supporting Information). In contrast, there were 611 up- and 627 downregulated genes in H1 ESCs after 48 hours of ACTIVIN A treatment (S2A Supporting Information). There were only 53 genes that were commonly upregulated between H9 and H1 ESCs after identical ACTIVIN A treatments, consistent with previous reports indicating large human ESC line differences [41, 42]. However, the 53 common upregulated genes were enriched for GO terms related to differentiation (S2B Supporting Information). There was greater concordance between the downregulated genes between H1 and H9 ESCs: 712 genes were downregulated in H9 ESCs after 48 hours, 627 were downregulated in H1 ESCs, and 112 of these were common to both cell lines (S3A Supporting Information). These common downregulated genes were significantly enriched for genes related to cation transport and localization (S3B Supporting Information).

ACTIVIN A treatment is thought to induce mesendoderm formation and the exit from pluripotency [8, 43]. This expected result was confirmed with the changes in morphology, loss of TRA-1-60, increase in CXCR4 staining, and general induction of differentiation transcription patterns (Fig 1A–1D). To further illustrate the induction of differentiation we indicated the change in expression for select germ layer- and pluripotency-specific markers within the volcano plot (Fig 1D). This result clearly shows that the majority of the germ-layer specific markers have increased expression levels between 6 and 48 hours of ACTIVIN A treatment (Fig 1D). For increased clarity, we have plotted the fold change for each germ layer marker from Fig 1D within individual bar plots (Fig 1E–1H. * p-adjusted < 0.1; Benjamini/Hochberg method [40]). Using this strict statistical significance threshold there were two significantly upregulated endoderm genes, GATA4 and SHH (Fig 1E); 1 significantly upregulated mesoderm gene, HAND1 (Fig 1F); no significantly upregulated ectoderm genes (Fig 1G); and a single significantly downregulated pluripotency marker, ALPL (Fig 1H). These data are consistent with our ACTIVIN A treatment inducing differentiation of H9 ESCs towards the mesendoderm lineage, with the appearance of specific markers occurring between 6 and 48 hours.

### Ribosomal RNA synthesis is significantly reduced within 6 hours of ACTIVIN A treatment in hESCs

**Fig 2:**
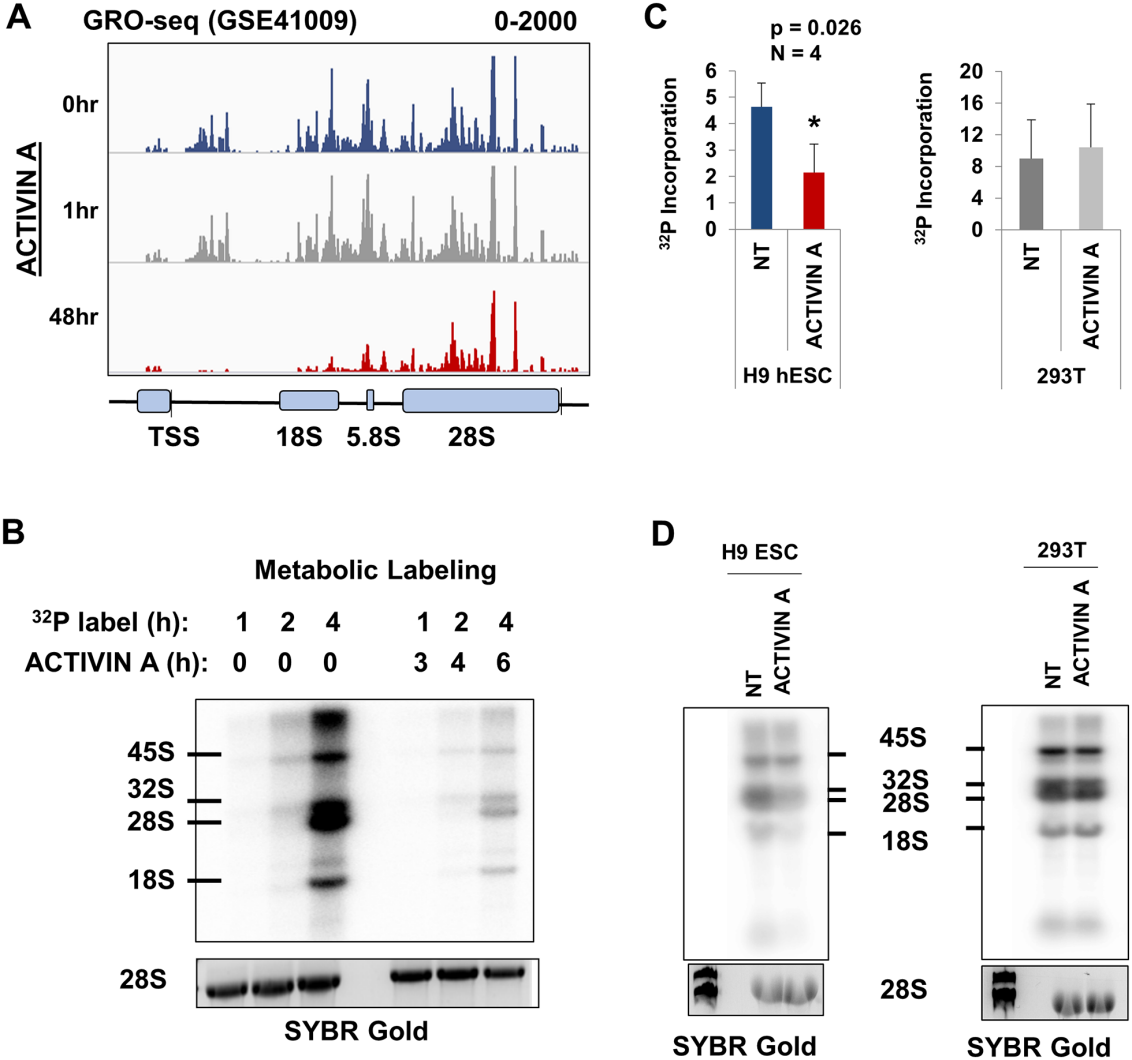
ACTIVIN A–induced differentiation coincides with rapid downregulation of rRNA synthesis. (A) The alignment of GRO-seq reads from GSE41009 to a consensus unit of the rRNA gene visualized using the Integrative Genomics Viewer [58]. The scale is set from 0 to 2000 reads per million. A schematic diagram of the rRNA gene is shown below the reads. The thin vertical line after the 28S region indicates the transcriptional termination site. (B) The metabolic labeling of total RNA from H9 ESCs either untreated or grown in ACTIVIN A (50 ng/ml) for the indicated length of time. Cells were first grown in phosphate-free media and then ^32^P was added (see Methods). A two hour time lag exists between the onset of ACTIVIN A treatment and the introduction of ^32^P. Total RNA was harvested and electrophoresed on a 1% agarose-formaldehyde denaturing gel. The RNA was transferred to a Zeta-Probe membrane and imaged using a phosphoimager. An identical gel was simultaneously stained with SYBR gold to visualize total RNA (shown below). The rRNA species is labeled to the left of the image. (C) The newly synthesized, radiolabeled, rRNA was quantified using the Typhoon phosphoimager, and then normalized to total RNA using the SYBR gold stain and imageJ software. The ratio of newly synthesized RNA to total RNA (28S rRNA) was then plotted after 6 hours of ACTIVIN A treatment (Student’s t-test p = 0.026, N = 4). A control experiment was carried out in an identical manner using HEK293T cells (gray bars). The y-axis represents counts per minute per pixel (see Methods). (D) Two representative gels, as described in (B), are lined up below their corresponding quantitation in (C).

Previous studies have indicated that the rate of rRNA synthesis is reduced concomitant with the differentiation of pluripotent stem cells [32, 34–36]. In this study, we wished to quantify the timing of the changes to rRNA synthesis rates during cellular differentiation. A previously published global run-on sequencing (GRO-seq) of H1 hESCs treated with ACTIVIN A was analyzed for the incorporation of the modified nucleoside into nascent rRNA by aligning reads to a consensus repeat of the human rRNA gene, as previously described (Methods) [37, 44]. This analysis demonstrated the distribution of Pol I within the rRNA gene (Fig 2A, top panel). There was a dearth of reads that aligned to the region in between the promoter and start of the 18S. The overall GRO-seq signal across the rRNA gene was unchanged after 1 hour of ACTIVIN A treatment, but was drastically lowered by 48 hours (Fig 2A).

GRO-seq is typically used to measure Pol II pausing and it has not been fully vetted as a mechanism to measure the overall activity of Pol I. Nevertheless, these results suggest that the rRNA synthesis rate was decreased within 48 hours of ACTIVIN A treatment. These data are also consistent with our observations that ESCs exit pluripotency and induce the transcription of differentiation markers within 48 hours (Fig 1). To validate the precise timing of the change in rRNA synthesis rate we performed metabolic labeling of H9 ESCs through a pulse of ^32^P inorganic phosphate (Methods). The labeling was done in both untreated and ACTIVIN A treated H9 ESCs. Total RNA was then harvested, electrophoresed, and the radiolabeled RNA was measured via GE Typhoon phosphorimager (Methods) (Fig 2B). The total Pol I activity during each treatment (untreated vs. ACTIVIN A) was measured by summing the radiolabeled observable intermediates of rRNA biogenesis (the 45S, 32S, 28S, and 18S rRNAs) and normalizing these counts to the total RNA (measured by quantifying SYBR gold staining of the identical gel using ImageJ, see Methods). After 4 hours of labeling (which corresponds to 6 hours of ACTIVIN A treatment), the total incorporation of rRNA is reduced by >50% (Fig 2B and 2C: 4.6 (c.p.m./pixel) vs. 2.1 (c.p.m./pixel), Student’s t-test, N = 4, p = 0.026). By contrast, there is no significant change in the synthesis rate of rRNA within ACTIVIN A treated HEK293T cells (Fig 2C: 9.0 (c.p.m./pixel) vs 10.4 (c.p.m./pixel), gray bars). Interestingly, the rRNA synthesis rate was much higher in the transformed HEK293T cells than in H9 ESCs (Fig 2D: 9.0 (c.p.m./pixel) vs 4.6 (c.p.m./pixel)). We conclude that the change in rRNA synthesis occurs within 6 hours of ACTIVIN A treatment, which precedes the changes to many other germ layer specific transcripts.

### The reduction of rRNA synthesis during endodermal-lineage specification is concomitant with a reduction in UBTF binding, not heterochromatin formation

**Fig 3:**
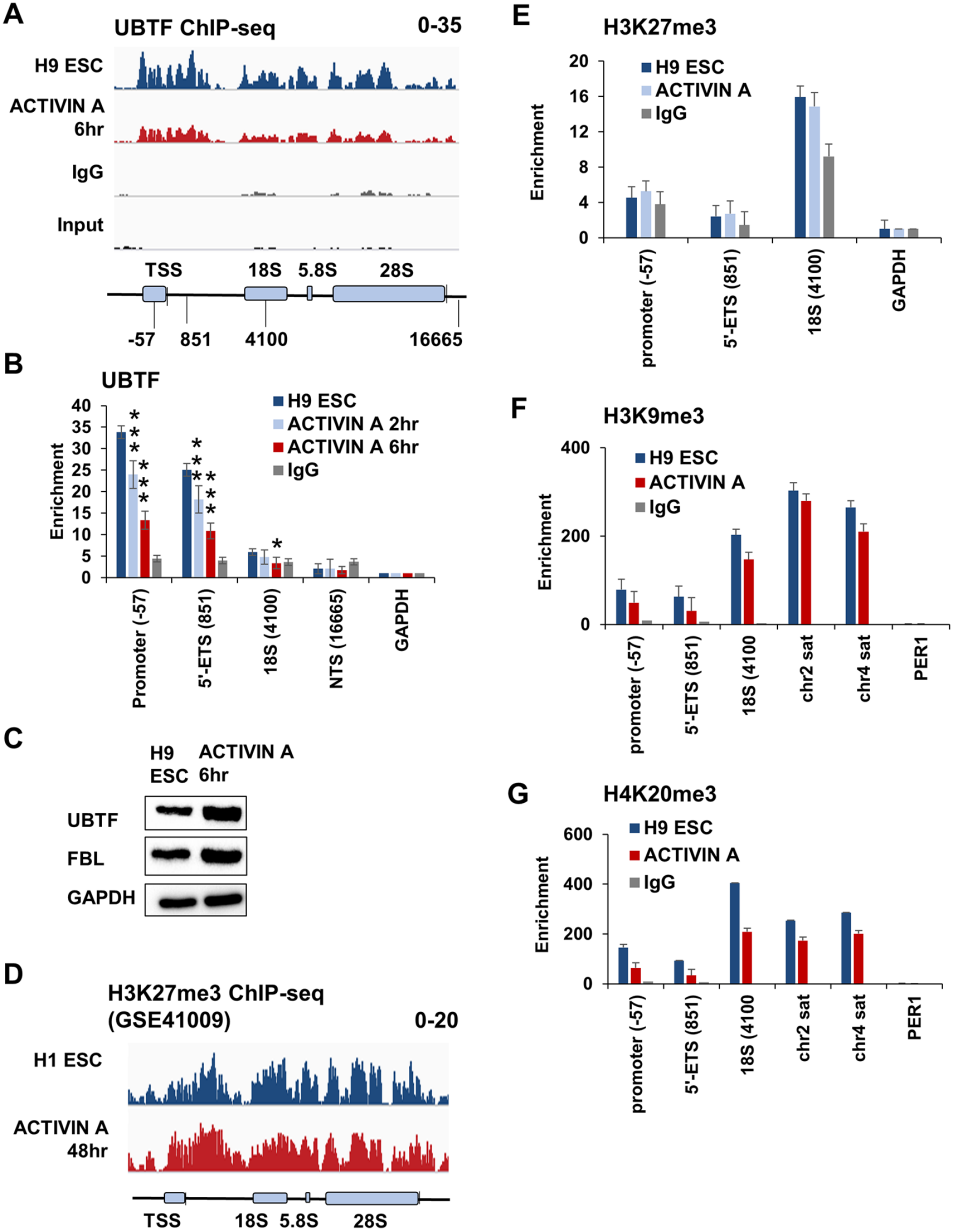
The reduction in rRNA gene synthesis is concomitant with dissociation of UBTF from the promoter, and precedes heterochromatin formation. (A) UBTF ChIP-seq from either untreated, or ACTIVIN A-treated H9 ESCs for 6 hours was aligned to a consensus unit of the human rRNA gene, and visualized as described in Fig 2A. (B) UBTF ChIP-QPCR across the indicated regions of the human rRNA gene. At each locus, the percent input of UBTF (and IgG) is shown. The percent input from each trial was then normalized to the percent input at *GAPDH*. The geometric mean is presented plus or minus the 95% confidence interval. Asterisks are used to indicate statistical significance (*** p < 0.001, * p < 0.05). The location of each primer set within the consensus rRNA gene is indicated beneath the schematic from (A) (5’-ETS = 5’-external transcribed spacer; NTS = non-transcribed spacer). (C) A Western blot of UBTF, Fibrillarin (FBL), and GAPDH in untreated and ACTIVIN A-treated H9 ESCs at 6 hours. (D) ChIP-seq of H3K27me3 (H1 ESCs: SRR212453; 48 hours ACTIVIN A treatment: SRR212471) was aligned to the consensus rRNA gene as described in Fig 2A [37]. (E) A ChIP-QPCR of H3K27me3 across the indicated regions of the rRNA gene in untreated and ACTIVIN A-treated H9 ESCs for 2 hours was calculated and presented as described in (B). ChIP-QPCR of H3K9me3 (F), and H4K20me3 (G) was carried out and presented as described in (B), but was done at 48 hours and normalized to *PER1*.

To compare the timing of the downregulation of rRNA synthesis with the binding of the Pol I transcription factor, UBTF, we performed ChIP-seq on untreated H9 ESCs and compared the binding to H9 ESCs after 6 hours of ACTIVIN A treatment (Fig 3A). The overall binding of UBTF to the rRNA gene was reduced roughly 50% after a 6 hour ACTIVIN A treatment. This was consistent with the reduction in total rRNA synthesis measured by ^32^P metabolic labeling (Fig 2). We confirmed the ChIP-seq result using ChIP-QPCR, and measured the dissociation at both 2 and 6 hours. We observed a significant reduction in UBTF enrichment at the promoter and 5’-ETS after only 2 hours ACTIVIN A treatment. We observed significant reductions at the promoter, 5’-ETS, and 18S regions after 6 hours, consistent with the ChIP-seq data (Fig 3B vs 3A, *** p < 0.001, * p < 0.05). The reduction in UBTF ChIP signal was not due to changes in UBTF transcript abundance (data not shown), or due to overall UBTF protein levels (Fig 3C).

Previous studies have shown that silencing of the rRNA gene in mouse ESCs (mESCs) is concomitant with increases to both H3K27me3 and H3K9me3 [32]. However, the earliest that these changes to heterochromatin formation are observed are after 7 days of differentiation from mESCs into Neural Precursor Cells (NPCs). The levels of various heterochromatin marks associated with the human rRNA gene (H3K27me3, H3K9me3, and H4K20me3) exhibit marked cell to cell variability [44], and the real-time changes to any heterochromatic histone modifications during hESC differentiation have not been investigated. To this end, we used publically available H3K27me3 ChIP-seq data to demonstrate that there is no change to H3K27me3 levels in H1 ESCs within 48 hours of ACTIVIN A treatment (Fig 3D, GSE41009). We confirmed the absence of earlier changes (2 hours) within H9 ESCs via ChIP-QPCR (Fig 3E). There were actually slight decreases in both H3K9me3 (Fig 3F) and H4K20me3 (Fig 3G) levels after 48 hours of ACTIVIN A treatment. We conclude that the dissociation of UBTF from the rRNA gene promoter occurs within 2 hours of ACTIVIN A treatment of H9 ESCs, and precedes heterochromatin formation.

### Direct inhibition of Pol I induces exit from pluripotency and differentiation

**Fig 4:**
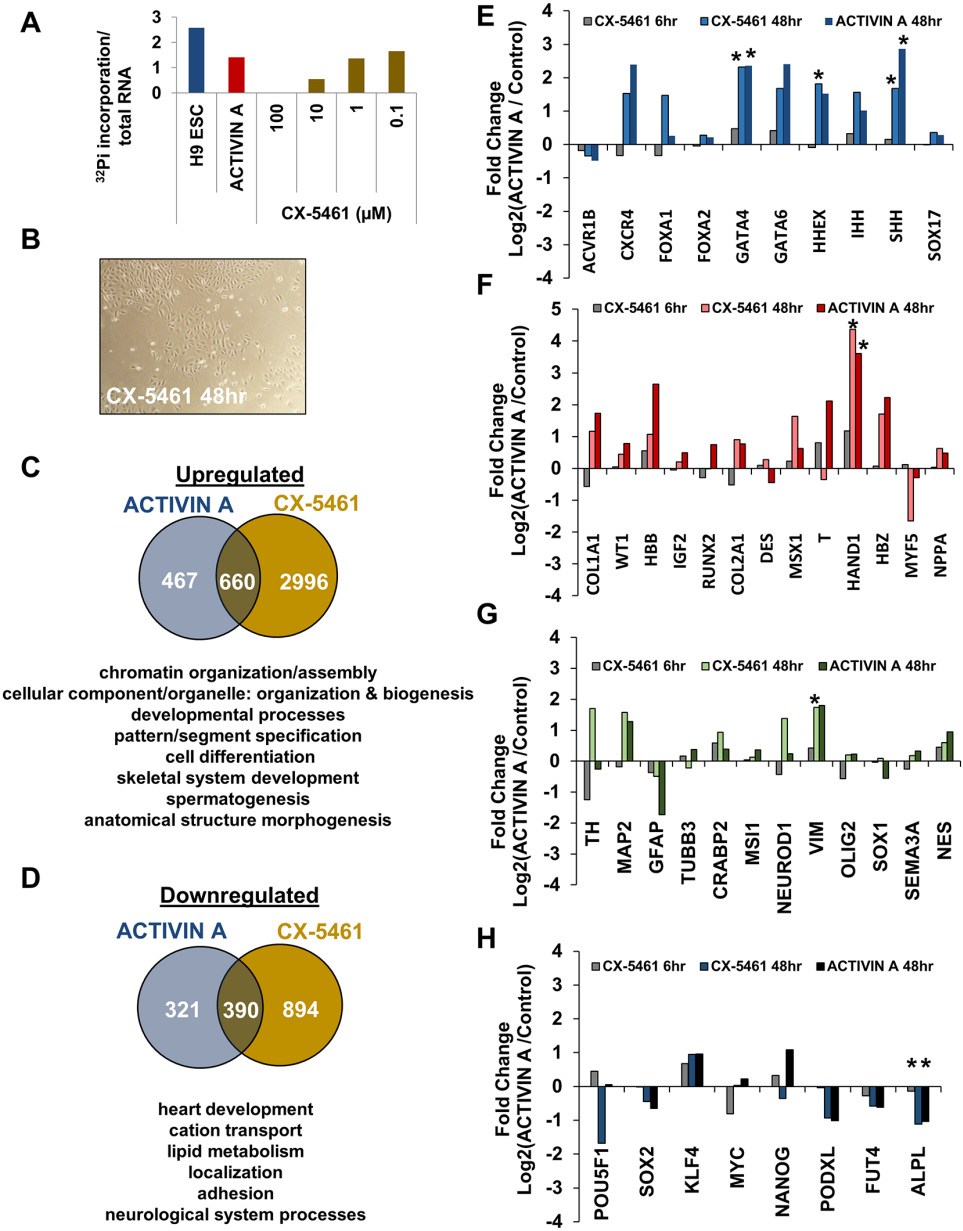
Direct inhibition of Pol I by CX-5461 induces loss of pluripotency and differentiation. (A) The metabolic labeling of H9 ESCs with ^32^P was carried out for 6 hours, as described in Fig 2B, but with the addition of a range of concentrations of CX-5461. The dose of CX-5461 is shown on the x-axis and the ratio of c.p.m (newly synthesized rRNA) to total 28S rRNA was calculated (see Methods) and plotted on the y-axis. (B) A phase-contrast image of CX-5461-treated H9 ESCs at 48 hours is shown. (C) A Venn diagram indicating the overlap between significantly upregulated genes after 48 hours of either ACTIVIN A or CX-5461 treatment. A list of the top PANTHER DB GO terms from the common genes is shown below. The full list is available in the Supporting Information. (D) The same analysis as described in (C) was performed for the downregulated genes. (E-H) The fold change in gene expression after 6 or 48 hours of CX-5461 treatment is shown for the same panel of genes from endoderm (E), mesoderm (F), ectoderm (G), and pluripotency-markers (H) that were depicted in Fig 1, alongside the fold change in gene expression after 48 hours of ACTIVIN A treatment (* p-adjusted < 0.1, Benjamini/Hochberg correction).

The reduction in both rRNA synthesis (Fig 2), and the partial dissociation of UBTF from chromatin within 6 hours of endodermal specification (Fig 3), occurred almost immediately upon exposure of H9 ESCs to ACTIVIN A. These observations were consistent with recent conclusions that the regulation of rRNA synthesis is an important factor in promoting differentiation [32, 34–36]. To test this hypothesis in our system, we utilized the Pol I inhibitor, CX-5461, to directly inhibit rRNA synthesis in H9 ESCs [45–48]. A titration of CX-5461 allowed for determination of a dose (1 μM), which reduced rRNA synthesis to an identical level as 50 ng/ml of ACTIVIN A treatment (Fig 4A). This treatment was sufficient to induce changes to H9 ESC colony morphology after 48 hours (Fig 4B). To determine how the direct reduction in rRNA gene transcription alters the cellular transcriptome in H9 ESCs we performed polyA+ RNA-seq in both 6 and 48 hour CX-5461 treated H9 ESCs. To identify genes that were significantly altered after CX-5461 treatment we performed a DESeq2 analysis along with the H1 and H9 RNA-seq samples described above (Fig 1, [40]). There were 83 significantly changed genes (45 up and 38 down) after 6 hours of CX-5461 treatment, which were not enriched for any particular GO term (S1 Supporting Information). However, after 48 hours of CX-5461 treatment there were 3389 upregulated (Fig 4C), and 1284 downregulated genes (Fig 4D). There were 660 upregulated (Fig 4C) and 390 downregulated genes in common (Fig 4D) between CX-5461- and ACTIVIN A-treated H9 ESCs. To compare the differentiation programs that were induced by each treatment, the up- and downregulated genes were input into a PANTHER GO analysis, as described above (Fig 1). The common upregulated genes were enriched for GO terms related to chromatin organization and cellular differentiation (Fig 4C). The common downregulated terms were related to cation transport, lipid metabolism, heart development, cellular adhesion, and neurological system development (Fig 4D). The full analysis of GO terms is shown within the supplemental information (S4 and S5 Supporting Information). CX-5461 and ACTIVIN A treatment had very similar effects on the expression of germ layer-specific markers (Fig 4E–4H, S4, and S5). CX-5461 treatment demonstrated a significant increase in 3 endodermal markers: GATA4, HHEX, and SHH (Fig 4E); 1 mesodermal marker: HAND1 (Fig 4F); 1 ectodermal marker: VIM (Fig 4G); and a significant decrease in 1 pluripotency marker: ALPL (Fig 4H). With the exceptions of VIM and HHEX, these significant changes were also observed after 48 hours of ACTIVIN A treatment.

## Discussion

There have been multiple reports indicating a correlation between the reduction in rRNA synthesis rate, the exit from pluripotency, and the differentiation of pluripotent stem cells [34–36]. Work in mESCs indicates that transcriptional silencing of the rRNA genes actively induces differentiation [32]. Here, we demonstrate that during the ACTIVIN A-induced directed differentiation of ESCs the reduction in rRNA synthesis rate occurs within 6 hours (Fig 2). This precedes significant changes to specific pluripotency- and germ layer-marker expression (Fig 1). The reduction in rRNA gene transcription occurs concomitantly with dissociation of UBTF from the rRNA gene, which is already significantly reduced by 2 hours of ACTIVIN A treatment (Fig 3).

The partial dissociation of UBTF occurs independent of changes to heterochromatin formation (Fig 3). Here we use three histone modifications as markers for heterochromatin formation at the rRNA gene, H3K27me3, H3K9me3, and H4K20me3. These modifications localize to the rRNA gene in a cell-type specific manner [44, 49], thus it was difficult to know *a priori* which would feature most prominently in this model system. There were no significant increases for any of these histone modifications within 48 hours of differentiation, despite changes to the rRNA synthesis rate occurring within 2–6 hours. We therefore conclude that the reduction in rRNA synthesis rate during the differentiation of H9 ESCs precedes heterochromatin formation. We did not investigate the role of DNA methylation for two reasons: the mosaic CpG methylation pattern within the human rRNA gene promoter does not easily correlate with expression [50], and CpG methylation typically follows heterochromatin formation during gene silencing [50, 51]. Previous studies have shown that CpG methylation is critical for preventing cryptic Pol II transcription at silent rRNA genes [52]. Future studies will be aimed at furthering our understanding of the timing and mechanistic role that CpG methylation plays in the developmental regulation of the rRNA gene.

We compared ACTIVIN A induced changes to both H1 and H9 hESC lines. These two cell lines have different transcription profiles and responses to ACTIVIN A. However, they both significantly upregulate differentiation-specific genes within 48 hours (S1 and S2 Supporting Information). Genes related to cation transport and localization were reduced in both cell lines. It remains unclear what role cation transport has in the process of differentiation. However, future studies will be directed at ablating the expression of these genes and determining the impact on both pluripotency and differentiation.

We have demonstrated that the Pol I specific inhibitor, CX-5461, induces differentiation of H9 ESCs at a dose that reduces the rRNA synthesis rate by 50%; equal to that achieved by 50 ng/ml of ACTIVIN A treatment (Fig 4). Previous studies that have artificially reduced the rRNA synthesis rate through disparate mechanisms (actinomycin D treatment [34], reduction in SL1 complex [34, 36], reduction in Fibrillarin [35], or initiating heterochromatin formation [32]) and all have observed a loss of pluripotency and induction of differentiation. Here we have addressed the mechanism by which this reduction in rRNA synthesis functions by performing RNA-seq at 6 and 48 hours post-treatment. The Pol I specific inhibitor does indeed induce the expression of markers from all three germ layers, and many of the upregulated genes were related to cellular differentiation. Greater than 50% of the induced (and repressed) genes after 48 hours of ACTIVIN A treatment in H9 ESCs were also induced (or repressed) after 48 hours of CX-5461 treatment, which indicates a much higher concordance between ACTIVIN A and CX-5461 treatment within H9 ESCs than between H1 and H9 ESCs after ACTIVIN A treatment. This similarity was also apparent when viewing the expression of specific germ-layer markers. All of the significantly affected genes after 48 hours of ACTIVIN A treatment (4) were also significantly changed upon CX-5461 treatment.

Despite the now large body of evidence that the regulation of rRNA gene transcription within stem cells is a critical aspect of cell fate determination, there still remains much work to understand the molecular basis for this relationship. We have advanced the understanding of this process by providing a timeline for important regulatory events during directed differentiation of H9 ESCs. Furthermore, the identification of differentially expressed genes in response to the reduction in rRNA gene transcription represents a first step in understanding this relationship. It is now clear that one of the early steps in differentiation of pluripotent stem cells is the downregulation of rRNA synthesis, and that this event can alone induce gene expression programs that closely mimic that of “natural” signaling molecules of the TGF-beta family. Future studies will be focused on two outstanding questions: 1) how is the transcription of the rRNA genes regulated within hESCs? and 2) how does this regulation impact cellular physiology to facilitate differentiation? The work here provides the first step in understanding these important processes.

## Materials and Methods

### Cell Culture and Drug Treatment

H9 ESCs were obtained from WiCell as per agreement 15-W0341. H9 ESCs were cultured on Matrigel coated plates (corning #354277) in mTeSR 1 media (STEMCELL Technologies # 05857), washed with DMEM/F12 (GE Healthcare # SH3002302) and passaged with accutase (MP Biomedicals # 091000449) and ROCK Inhibitor Y-27632 (BD # 562822). For ACTIVIN A treatment, cells were grown to about 75–80% confluency. The cells were washed with PBS and 10 ml of RPMI-1640 (GE Healthcare # SH30027.01) was added with 1X B-27 supplement (Life Technologies # 17504–044) along with ACTIVIN A (Sigma # SRP3003) to a final concentration of 50 ng/ml. For CX-5461 treatment the cells were washed with PBS and received RPMI with B-27 as above with CX-5461 (Selleckchem # 52684) to a final concentration of 1 μM. The media was replaced daily with fresh reagents until cells were harvested. HEK293T cells were maintained in DMEM with high glucose (GE Healthcare # SH30243.01) with 10% FBS (GE Healthcare # SH30070.03) and passaged with 0.25% trypsin-EDTA (Gibco # 25200–056).

### Metabolic Labeling

For ^32^P labeling cells were grown in the presence of drug for 2 hours. 20 μl of phosphorus-32 radionuclide [^32^P] 1 mCi/ml (Perkin Elmer # NEX053001MC) was then added to each plate, along with serum-free, phosphate-free RPMI-1640 with B-27, mixed well, and were incubated an additional 1, 2, or 4 hours before being harvested with TRIzol (Life Technologies # 10296028). The RNA was further isolated with chloroform extraction and ethanol precipitation. The RNA was electrophoresed on a 1% formaldehyde/MOPS agarose gel, stained with SYBR gold (Life Technologies # S-11494), and imaged with a Bio-Rad chemi doc MP imaging system. The 18S and 28S bands were quantified with imageJ. The RNA was then transferred overnight with 10X SSC to a Zeta-Probe blotting membrane (Bio-Rad # 162–0165). The membrane was exposed to a phosphoscreen (GE) and imaged using a Typhoon Trio + imager (GE). The rate of rRNA synthesis was calculated by adjusting the intensity of the radiolabeled species for exposure time and specific activity of the ^32^P (as per the assay date). This adjusted signal intensity was then divided by pixel intensity of the total rRNA signal, which was also adjusted for exposure time.

### TRA-1-60 Staining

Human ESCs were cultured with or without ACTIVIN A (50ng/ml) or CX-5461 (1 μM) for 48 hours, washed with PBS and harvested with 0.25% trypsin-EDTA. Approximately 200,000–400,000 cells were stained on ice with anti-TRA-1-60 antibody (1:100, Santa Cruz Biotechnology sc-21705) for 1 hour and FITC conjugated secondary antibody (1:300, BD # 555988) for 30 minutes. The cells were then stained with 1 μg/ml propidium iodide (PI) at room temperature for 5 minutes before being analyzed on a LSR Fortessa Analyzer (BD). Dead cells that were stained PI positive were excluded from further analysis. Cells stained only with the secondary antibody were used to set up the TRA-1-60 negative gate. Data were analyzed by the BD FACSDiva software.

### Immunofluorescence

For immunostaining, untreated and ACTIVIN A treated H9 ESCs (48 hours) were fixed in 4% paraformaldehyde, blocked in Protein Block (Dako), and incubated with CXCR4 antibody (BioLegend) overnight at 4°C followed by Alexa Fluor 488-conjugated secondary antibody (Thermo Fisher) for 2 hours at room temperature. Nuclei were stained with DAPI. Images were acquired by a Nikon Ti-S microscope.

### ChIP

ChIP was performed as previously described but using 1.2% formaldehyde [53]. Immunoprecipitations were performed with the following antibodies: anti-UBTF (Millipore # MABE195), anti-H3K27me3 (Millipore # 07–449), anti-H3K9me3 (Abcam ab8898), and anti-H4K20me3 (ab9053). Samples were either used for sequencing on an Illumina Hi-seq or used for QPCR with SYBR Select master mix (ABI # 4472897). ChIP-QPCR data was plotted as the percent input, which was then normalized relative to a negative control locus (either *GAPDH* or *PER1*, see “Primer Sequences”).

### Western Blot

Western blotting was performed as previously described [53], using anti-UBTF (Millipore # MABE195), anti-FBL (Santa Cruz #sc-166001), and anti-GAPDH (Abcam ab128915) antibodies.

### RNA-seq

The poly A+ RNA-seq (library prep and sequencing) was performed by Hudson Alpha Institute for Biotechnology). Briefly, total RNA was polyA+ purified two times, converted to cDNA, and then adapters were added using the Illumina TruSeq v.2 kit.

### Primer Sequences (all sequences 5’ to 3’)

-57: F-CCCGGGGGAGGTATATCTTT; R-ACAGGTCGCCAGAGGACAG

5’-ETS (851): F-GAACGGTGGTGTGTCGTTC; R-CGTCTCGTCTCGTCTCACTC

18S (4100): F- GAGAAACGGCTACCACATCC; R- CAATTACAGGGCCTCGAAAG

NTS (16665): F- GATGGGTTTCGGGGTTCTAT; R- GGCAGGCAAATGTAGAAGGA

GAPDH: F- TACTAGCGGTTTTACGGGCG; R: TCGAACAGGAGGAGCAGAGAGCGA

PER1: GGAGTGCCCCCACAATGATTA; R: TCCCTGGAAACGTGGGTAGT

### Data analysis

#### DESeq2

RNA-seq was aligned to hg19 using TopHat [54] with the –b2-very-fast and–no-coverage-search options used. The aligned BAM files were converted to BED using BEDTools [55] *bamToBed* with the–split option. The BED files were then intersected with a non-redundant list of genes in hg19, which was created by grouping each gene name using BEDTools *groupBy*. The intersect was done using *intersectBed* with the–c option, which counts the number of RNA-seq tags that align within each gene. This was done for each of all RNA-seq datasets in this manuscript, untreated H9 ESCs (GSM2028278; GSM2175118; GSM2175119), 6 hour ACTIVIN A (GSM2028279; GSM2175120; GSM2175121), 6 hour CX-5461 (GSM2028280; GSM2175122; GSM2175123), 48 hour ACTIVIN A (GSM2028281; GSM2175124; GSM2175125; GSM2175126), and 48 hour CX-5461 (GSM2028282; GSM2175127; GSM2175128; GSM2175129) as well as for previously published H1 RNA-seq datasets, untreated H1 (GSM1006724; GSM1006725) and 48 hour ACTIVIN A H1 (GSM1006726; GSM1006727). This data was next placed in a countTable format which shows the total coverage across 42,785 unique genes (rows) for each dataset (columns). This was input into DESeq2 within R studio for differential expression analysis, using a Benjamini/Hochberg corrected p-value cut-off of 0.1 (p-adjusted) [40]. The scatterplot was generated using R studio. The countTable is shown in S1 Supporting Information.

#### GRO-seq

All replicates for the 0 hour, 1 hour, and 48 hour datasets for GRO-seq were downloaded from the Genome Expression Omnibus (GEO) as SRA files corresponding to sample sets GSM1006729, GSM1006730, and GSM1006731, respectively. All SRA files were converted to FASTQ files using *fastq-dump*.*2* tool from the SRA Toolkit. FASTQ files for all replicates of each time point were concatenated together. Concatenated FASTQ files were aligned using *bowtie2* with default parameters to both hg19 (for analysis outside of the rRNA gene) and a previously described custom genome which includes the rRNA gene consensus as an extra chromosome (for the rRNA gene analysis) [56]. Aligned tags in SAM format were converted to BAM using SAMtools. BAM files were converted to BED using *bamToBed* tool from BEDtools. Strand-specific reads were selected using the BEDtools suite.

#### ChIP-seq

FASTQ files were quality filtered using *fastq_quality_filter* from FASTX-Toolkit with the “-q” parameter set at 20 and the “-p” parameter set at 80. Quality filtered FASTQ files were then collapsed to help eliminate PCR bias using the *fastx_collapser* tool from FASTX-Toolkit with default parameters. The resulting FASTA files were aligned using *bowtie2* with default parameters to a previously described custom genome which includes the rRNA gene consensus as an extra chromosome [56]. Aligned tags in SAM format were converted to BAM using SAMtools. BAM files were converted to BED using *bamToBed* tool from BEDtools.

#### GO analysis

The PANTHER DB was used for all GO analyses. The list of up- or downregulated gene names was uploaded into the PANTHER DB server, and a statistical overrepresentation analysis was done for “Biological processes, and PANTHER pathways”. The overlap between genes within each RNA-seq dataset were determined using the *join* command in UNIX, and presented as Venn diagrams that were created in R studio.

### Data acquisition

All acquired datasets were downloaded from the Genome Expression Omnibus (GEO) [57]. GRO-seq data for 0 hour, 1 hour, and 48 hour time points were sample sets GSM1006729, GSM1006730, and GSM1006731, respectively. H1 RNA-seq at 0 hour and 48 hour time points were samples sets GSM1006724/GSM1006725 and GSM1006726/GSM1006727, respectively. The UBTF ChIP-seq and all RNA-seq datasets generated in this study can be accessed through the Gene Expression Omnibus (GEO) using the accession number GSE76586.

## Supporting Information

S1 Supporting Information A summary of RNA-seq and PANTHER GO analysis. (A) This tab represents the countTable summary from all RNA-seq datasets analyzed in the manuscript. The countTable was generated using BEDtools, as described in the methods. The countTable is the primary data that is input into DESeq2 for downstream analysis. (B-F) These tabs represent the results output file from DESeq2 for each pair-wise comparison. Each comparison was made relative to untreated line-matched ESCs.
(XLSX)

S2 Supporting Information (A) A Venn diagram showing the differences in the genes that were upregulated in either H9 or H1 ESCs after 48 hours of ACTIVIN A treatment. (B) A PANTHER DB GO analysis for PANTHER Slim Biological Processes was performed on the 53 genes that were significantly upregulated after 48 hours of ACTIVIN A treatment in both H1 and H9 ESCs. The significance cutoff was a p-adjusted < 0.1, using the Bonferroni correction. The horizontal axis represents the–log_10_ of the adjusted p-value.
(TIF)

S3 Supporting Information (A) A Venn diagram showing the overlap between significantly downregulated genes after 48 hours of ACTIVIN A treatment in either H9 or H1 ESCs. (B) A PANTHER GO analysis for Slim-Biological processes was performed on the common downregulated genes. The significance cutoff was a p-adjusted < 0.05, using the Bonferroni correction. The–log_10_ p-value for over-representation is shown for the GO terms that were common to both cell lines.
(TIF)

S4 Supporting Information A PANTHER DB GO analysis for PANTHER Slim Biological Processes was performed on all significantly upregulated genes after 48 hours of either ACTIVIN A or CX-5461 treatment on H9 ESCs. (A) GO terms that were enriched in both ACTIVIN A and CX-5461 treatments. (B) GO terms that were enriched only after ACTIVIN A treatment. (C) GO terms that were enriched only after CX-5461 treatment. The variable axis represents the–log_10_ adjusted p-value of the statistical over-representation of each term, as computed by the PANTHER DB using the Bonferroni correction for multiple hypothesis testing.
(TIF)

S5 Supporting Information A PANTHER DB GO analysis for PANTHER Slim Biological Processes was performed on all significantly downregulated genes after 48 hours of either ACTIVIN A or CX-5461 treatment on H9 ESCs. (A) GO terms that were enriched in both ACTIVIN A and CX-5461 treatments. (B) GO terms that were enriched only after ACTIVIN A treatment. (C) GO terms that were enriched only after CX-5461 treatment. The variable axis represents the–log_10_ adjusted p-value of the statistical over-representation of each term, as computed by the PANTHER DB using the Bonferroni correction for multiple hypothesis testing.
(TIF)

## References

[1] Young RA. Control of the embryonic stem cell state. Cell. 2011;144(6):940–54. 10.1016/j.cell.2011.01.032 21414485 PMC3099475

[2] Gaspar-Maia A, Alajem A, Meshorer E, Ramalho-Santos M. Open chromatin in pluripotency and reprogramming. Nature reviews Molecular cell biology. 2011;12(1):36–47. 10.1038/nrm3036 21179060 PMC3891572

[3] Meshorer E, Misteli T. Chromatin in pluripotent embryonic stem cells and differentiation. Nature reviews Molecular cell biology. 2006;7(7):540–6. 10.1038/nrm1938 . 16723974

[4] Meshorer E, Yellajoshula D, George E, Scambler PJ, Brown DT, Misteli T. Hyperdynamic plasticity of chromatin proteins in pluripotent embryonic stem cells. Developmental cell. 2006;10(1):105–16. 10.1016/j.devcel.2005.10.017 16399082 PMC1868458

[5] Bhattacharya D, Talwar S, Mazumder A, Shivashankar GV. Spatio-temporal plasticity in chromatin organization in mouse cell differentiation and during Drosophila embryogenesis. Biophysical journal. 2009;96(9):3832–9. 10.1016/j.bpj.2008.11.075 19413989 PMC3297759

[6] Efroni S, Duttagupta R, Cheng J, Dehghani H, Hoeppner DJ, Dash C, et al Global transcription in pluripotent embryonic stem cells. Cell stem cell. 2008;2(5):437–47. 10.1016/j.stem.2008.03.021 18462694 PMC2435228

[7] Boles NC, Hirsch SE, Le S, Corneo B, Najm F, Minotti AP, et al NPTX1 regulates neural lineage specification from human pluripotent stem cells. Cell reports. 2014;6(4):724–36. 10.1016/j.celrep.2014.01.026 . 24529709

[8] Ikonomou L, Kotton DN. Derivation of endodermal progenitors from pluripotent stem cells. Journal of cellular physiology. 2014 10.1002/jcp.24771 25160562 PMC4344429

[9] Noseda M, Peterkin T, Simoes FC, Patient R, Schneider MD. Cardiopoietic factors: extracellular signals for cardiac lineage commitment. Circulation research. 2011;108(1):129–52. 10.1161/CIRCRESAHA.110.223792 . 21212394

[10] Shaltouki A, Peng J, Liu Q, Rao MS, Zeng X. Efficient generation of astrocytes from human pluripotent stem cells in defined conditions. Stem cells. 2013;31(5):941–52. 10.1002/stem.1334 . 23341249

[11] McStay B, Grummt I. The epigenetics of rRNA genes: from molecular to chromosome biology. Annual review of cell and developmental biology. 2008;24:131–57. 10.1146/annurev.cellbio.24.110707.175259 . 18616426

[12] Learned RM, Learned TK, Haltiner MM, Tjian RT. Human rRNA transcription is modulated by the coordinate binding of two factors to an upstream control element. Cell. 1986;45(6):847–57. . 3708692 10.1016/0092-8674(86)90559-3

[13] Learned RM, Cordes S, Tjian R. Purification and characterization of a transcription factor that confers promoter specificity to human RNA polymerase I. Molecular and cellular biology. 1985;5(6):1358–69. 3929071 10.1128/mcb.5.6.1358-1369.1985 PMC366865

[14] Bell SP, Learned RM, Jantzen HM, Tjian R. Functional cooperativity between transcription factors UBF1 and SL1 mediates human ribosomal RNA synthesis. Science. 1988;241(4870):1192–7. . 3413483 10.1126/science.3413483

[15] Clos J, Buttgereit D, Grummt I. A purified transcription factor (TIF-IB) binds to essential sequences of the mouse rDNA promoter. Proceedings of the National Academy of Sciences of the United States of America. 1986;83(3):604–8. 3456157 10.1073/pnas.83.3.604 PMC322912

[16] Cavanaugh AH, Evans A, Rothblum LI. Mammalian Rrn3 is required for the formation of a transcription competent preinitiation complex containing RNA polymerase I. Gene expression. 2008;14(3):131–47. 18590050 PMC2526047

[17] Moorefield B, Greene EA, Reeder RH. RNA polymerase I transcription factor Rrn3 is functionally conserved between yeast and human. Proceedings of the National Academy of Sciences of the United States of America. 2000;97(9):4724–9. 10.1073/pnas.080063997 10758157 PMC18300

[18] Yamamoto RT, Nogi Y, Dodd JA, Nomura M. RRN3 gene of Saccharomyces cerevisiae encodes an essential RNA polymerase I transcription factor which interacts with the polymerase independently of DNA template. The EMBO journal. 1996;15(15):3964–73. 8670901 PMC452116

[19] Panov KI, Friedrich JK, Russell J, Zomerdijk JC. UBF activates RNA polymerase I transcription by stimulating promoter escape. The EMBO journal. 2006;25(14):3310–22. 10.1038/sj.emboj.7601221 16858408 PMC1523182

[20] Stefanovsky V, Langlois F, Gagnon-Kugler T, Rothblum LI, Moss T. Growth factor signaling regulates elongation of RNA polymerase I transcription in mammals via UBF phosphorylation and r-chromatin remodeling. Molecular cell. 2006;21(5):629–39. 10.1016/j.molcel.2006.01.023 . 16507361

[21] Sanij E, Hannan RD. The role of UBF in regulating the structure and dynamics of transcriptionally active rDNA chromatin. Epigenetics: official journal of the DNA Methylation Society. 2009;4(6):374–82. . 19717978 10.4161/epi.4.6.9449

[22] Morgan GT, Reeder RH, Bakken AH. Transcription in cloned spacers of Xenopus laevis ribosomal DNA. Proceedings of the National Academy of Sciences of the United States of America. 1983;80(21):6490–4. 6579535 10.1073/pnas.80.21.6490 PMC390139

[23] McKnight SL, Miller OL Jr. Ultrastructural patterns of RNA synthesis during early embryogenesis of Drosophila melanogaster. Cell. 1976;8(2):305–19. . 822943 10.1016/0092-8674(76)90014-3

[24] Hamperl S, Wittner M, Babl V, Perez-Fernandez J, Tschochner H, Griesenbeck J. Chromatin states at ribosomal DNA loci. Biochimica et biophysica acta. 2013;1829(3–4):405–17. 10.1016/j.bbagrm.2012.12.007 . 23291532

[25] Conconi A, Widmer RM, Koller T, Sogo JM. Two different chromatin structures coexist in ribosomal RNA genes throughout the cell cycle. Cell. 1989;57(5):753–61. . 2720786 10.1016/0092-8674(89)90790-3

[26] Strohner R, Nemeth A, Nightingale KP, Grummt I, Becker PB, Langst G. Recruitment of the nucleolar remodeling complex NoRC establishes ribosomal DNA silencing in chromatin. Molecular and cellular biology. 2004;24(4):1791–8. 14749393 10.1128/MCB.24.4.1791-1798.2004 PMC344174

[27] Santoro R, Grummt I. Molecular mechanisms mediating methylation-dependent silencing of ribosomal gene transcription. Molecular cell. 2001;8(3):719–25. . 11583633 10.1016/s1097-2765(01)00317-3

[28] Bird AP, Taggart MH, Gehring CA. Methylated and unmethylated ribosomal RNA genes in the mouse. Journal of molecular biology. 1981;152(1):1–17. . 6279862 10.1016/0022-2836(81)90092-9

[29] Santoro R, Schmitz KM, Sandoval J, Grummt I. Intergenic transcripts originating from a subclass of ribosomal DNA repeats silence ribosomal RNA genes in trans. EMBO reports. 2010;11(1):52–8. 10.1038/embor.2009.254 20010804 PMC2816622

[30] Stefanovsky V, Moss T. Regulation of rRNA synthesis in human and mouse cells is not determined by changes in active gene count. Cell cycle. 2006;5(7):735–9. . 16582637 10.4161/cc.5.7.2633

[31] Poortinga G, Wall M, Sanij E, Siwicki K, Ellul J, Brown D, et al c-MYC coordinately regulates ribosomal gene chromatin remodeling and Pol I availability during granulocyte differentiation. Nucleic acids research. 2011;39(8):3267–81. 10.1093/nar/gkq1205 21177653 PMC3082905

[32] Savic N, Bar D, Leone S, Frommel SC, Weber FA, Vollenweider E, et al lncRNA maturation to initiate heterochromatin formation in the nucleolus is required for exit from pluripotency in ESCs. Cell stem cell. 2014;15(6):720–34. 10.1016/j.stem.2014.10.005 . 25479748

[33] Schlesinger S, Selig S, Bergman Y, Cedar H. Allelic inactivation of rDNA loci. Genes & development. 2009;23(20):2437–47. 10.1101/gad.544509 19833769 PMC2764490

[34] Hayashi Y, Kuroda T, Kishimoto H, Wang C, Iwama A, Kimura K. Downregulation of rRNA transcription triggers cell differentiation. PloS one. 2014;9(5):e98586 10.1371/journal.pone.0098586 24879416 PMC4039485

[35] Watanabe-Susaki K, Takada H, Enomoto K, Miwata K, Ishimine H, Intoh A, et al Biosynthesis of ribosomal RNA in nucleoli regulates pluripotency and differentiation ability of pluripotent stem cells. Stem cells. 2014;32(12):3099–111. 10.1002/stem.1825 . 25187421

[36] Zhang Q, Shalaby NA, Buszczak M. Changes in rRNA transcription influence proliferation and cell fate within a stem cell lineage. Science. 2014;343(6168):298–301. 10.1126/science.1246384 24436420 PMC4084784

[37] Sigova AA, Mullen AC, Molinie B, Gupta S, Orlando DA, Guenther MG, et al Divergent transcription of long noncoding RNA/mRNA gene pairs in embryonic stem cells. Proceedings of the National Academy of Sciences of the United States of America. 2013;110(8):2876–81. 10.1073/pnas.1221904110 23382218 PMC3581948

[38] Hou J, Charters AM, Lee SC, Zhao Y, Wu MK, Jones SJ, et al A systematic screen for genes expressed in definitive endoderm by Serial Analysis of Gene Expression (SAGE). BMC developmental biology. 2007;7:92 10.1186/1471-213X-7-92 17683524 PMC1950885

[39] Li F, He Z, Li Y, Liu P, Chen F, Wang M, et al Combined activin A/LiCl/Noggin treatment improves production of mouse embryonic stem cell-derived definitive endoderm cells. Journal of cellular biochemistry. 2011;112(4):1022–34. 10.1002/jcb.22962 . 21400570

[40] Love MI, Huber W, Anders S. Moderated estimation of fold change and dispersion for RNA-seq data with DESeq2. Genome biology. 2014;15(12):550 10.1186/s13059-014-0550-8 25516281 PMC4302049

[41] Osafune K, Caron L, Borowiak M, Martinez RJ, Fitz-Gerald CS, Sato Y, et al Marked differences in differentiation propensity among human embryonic stem cell lines. Nature biotechnology. 2008;26(3):313–5. 10.1038/nbt1383 . 18278034

[42] Allegrucci C, Young LE. Differences between human embryonic stem cell lines. Hum Reprod Update. 2007;13(2):103–20. 10.1093/humupd/dml041 . 16936306

[43] Lee HJ, Seo GY, Kim JH, Lee MR, Kim PH. Activin A stimulates mouse macrophages to express APRIL via the Smad3 and ERK/CREB pathways. Immunology letters. 2011;140(1–2):92–6. 10.1016/j.imlet.2011.07.001 . 21784102

[44] Zentner GE, Saiakhova A, Manaenkov P, Adams MD, Scacheri PC. Integrative genomic analysis of human ribosomal DNA. Nucleic acids research. 2011;39(12):4949–60. 10.1093/nar/gkq1326 21355038 PMC3130253

[45] Negi SS, Brown P. rRNA synthesis inhibitor, CX-5461, activates ATM/ATR pathway in acute lymphoblastic leukemia, arrests cells in G2 phase and induces apoptosis. Oncotarget. 2015 . 26061708 10.18632/oncotarget.4093 PMC4627237

[46] Haddach M, Schwaebe MK, Michaux J, Nagasawa J, O'Brien SE, Whitten JP, et al Discovery of CX-5461, the First Direct and Selective Inhibitor of RNA Polymerase I, for Cancer Therapeutics. ACS Med Chem Lett. 2012;3(7):602–6. 10.1021/ml300110s 24900516 PMC4025669

[47] Drygin D, Lin A, Bliesath J, Ho CB, O'Brien SE, Proffitt C, et al Targeting RNA polymerase I with an oral small molecule CX-5461 inhibits ribosomal RNA synthesis and solid tumor growth. Cancer research. 2011;71(4):1418–30. 10.1158/0008-5472.CAN-10-1728 . 21159662

[48] CX-5461 inhibits RNA Pol I in blood cancers. Cancer discovery. 2014;4(12):OF5 10.1158/2159-8290.CD-NB2014-154 . 25477122

[49] Zentner GE, Balow SA, Scacheri PC. Genomic Characterization of the Mouse Ribosomal DNA Locus. G3. 2013 10.1534/g3.113.009290 . 24347625 PMC3931559

[50] Ghoshal K, Majumder S, Datta J, Motiwala T, Bai S, Sharma SM, et al Role of human ribosomal RNA (rRNA) promoter methylation and of methyl-CpG-binding protein MBD2 in the suppression of rRNA gene expression. The Journal of biological chemistry. 2004;279(8):6783–93. 10.1074/jbc.M309393200 14610093 PMC2242730

[51] Hawkins PG, Santoso S, Adams C, Anest V, Morris KV. Promoter targeted small RNAs induce long-term transcriptional gene silencing in human cells. Nucleic acids research. 2009;37(9):2984–95. 10.1093/nar/gkp127 19304753 PMC2685082

[52] Gagnon-Kugler T, Langlois F, Stefanovsky V, Lessard F, Moss T. Loss of human ribosomal gene CpG methylation enhances cryptic RNA polymerase II transcription and disrupts ribosomal RNA processing. Molecular cell. 2009;35(4):414–25. 10.1016/j.molcel.2009.07.008 . 19716787

[53] Woolnough JL, Atwood BL, Giles KE. Argonaute 2 binds directly to tRNA genes and promotes gene repression in cis. Molecular and cellular biology. 2015 10.1128/MCB.00076-15 . 25918241 PMC4456445

[54] Trapnell C, Roberts A, Goff L, Pertea G, Kim D, Kelley DR, et al Differential gene and transcript expression analysis of RNA-seq experiments with TopHat and Cufflinks. Nature protocols. 2012;7(3):562–78. 10.1038/nprot.2012.016 22383036 PMC3334321

[55] Quinlan AR, Hall IM. BEDTools: a flexible suite of utilities for comparing genomic features. Bioinformatics. 2010;26(6):841–2. 10.1093/bioinformatics/btq033 20110278 PMC2832824

[56] van de Nobelen S, Rosa-Garrido M, Leers J, Heath H, Soochit W, Joosen L, et al CTCF regulates the local epigenetic state of ribosomal DNA repeats. Epigenetics & chromatin. 2010;3(1):19 Epub 2010/11/10. 10.1186/1756-8935-3-19 21059229 PMC2993708

[57] Edgar R, Domrachev M, Lash AE. Gene Expression Omnibus: NCBI gene expression and hybridization array data repository. Nucleic acids research. 2002;30(1):207–10. 11752295 10.1093/nar/30.1.207 PMC99122

[58] Robinson JT, Thorvaldsdottir H, Winckler W, Guttman M, Lander ES, Getz G, et al Integrative genomics viewer. Nature biotechnology. 2011;29(1):24–6. 10.1038/nbt.1754 21221095 PMC3346182

