# Supplementary figures and images for: The Regulation of rRNA Gene Transcription during Directed Differentiation of Human Embryonic Stem Cells
Source: PLoS One. 2016 Jun 14;11(6):e0157276. doi: 10.1371/journal.pone.0157276 (PMC4907514; doi:10.1371/journal.pone.0157276)

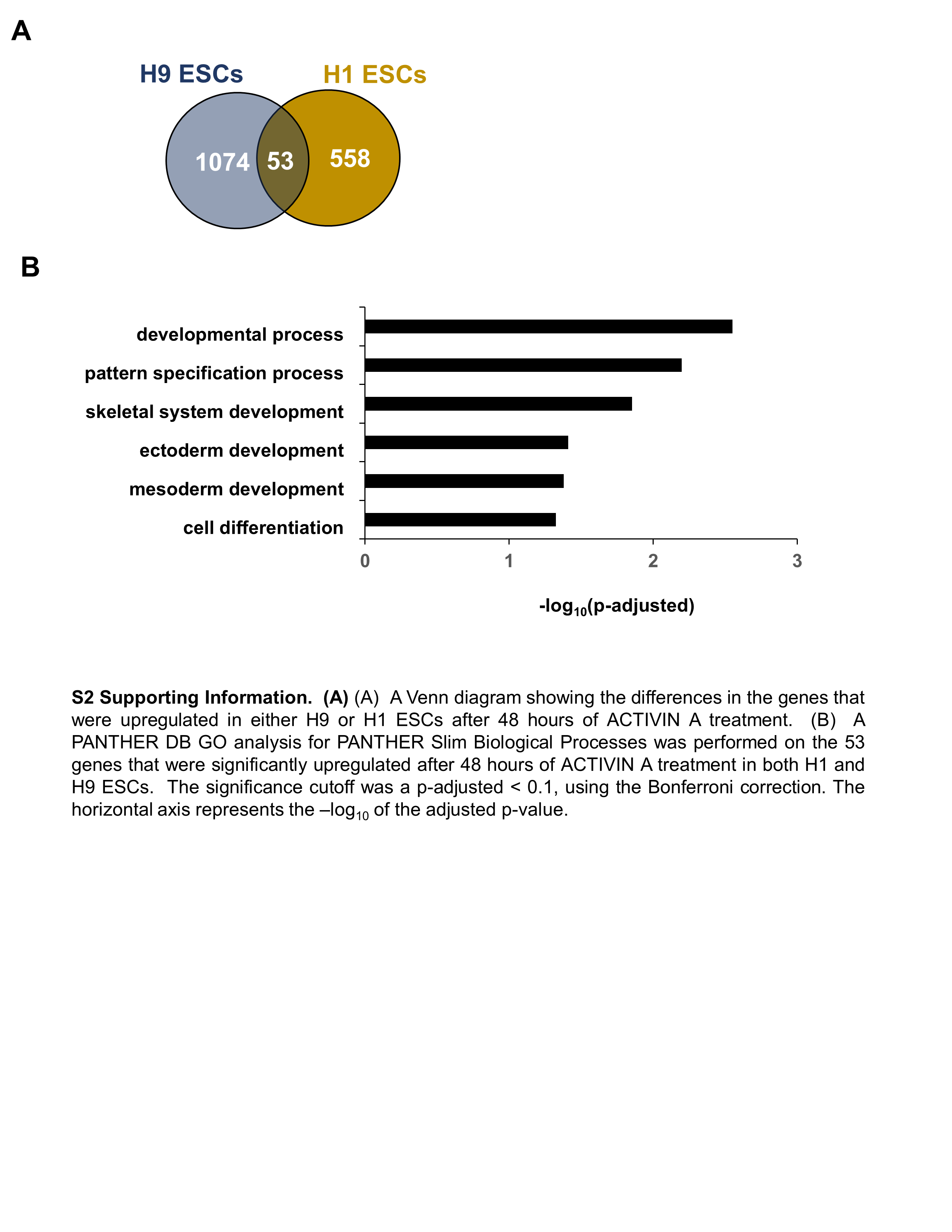

Supplement: S2 Supporting Information — (A) A Venn diagram showing the differences in the genes that were upregulated in either H9 or H1 ESCs after 48 hours of ACTIVIN A treatment. (B) A PANTHER DB GO analysis for PANTHER Slim Biological Processes was performed on the 53 genes that were significantly upregulated after 48 hours of ACTIVIN A treatment in both H1 and H9 ESCs. The significance cutoff was a p-adjusted < 0.1, using the Bonferroni correction. The horizontal axis represents the–log10 of the adjusted p-value. (TIF) [file pone.0157276.s002.TIF]

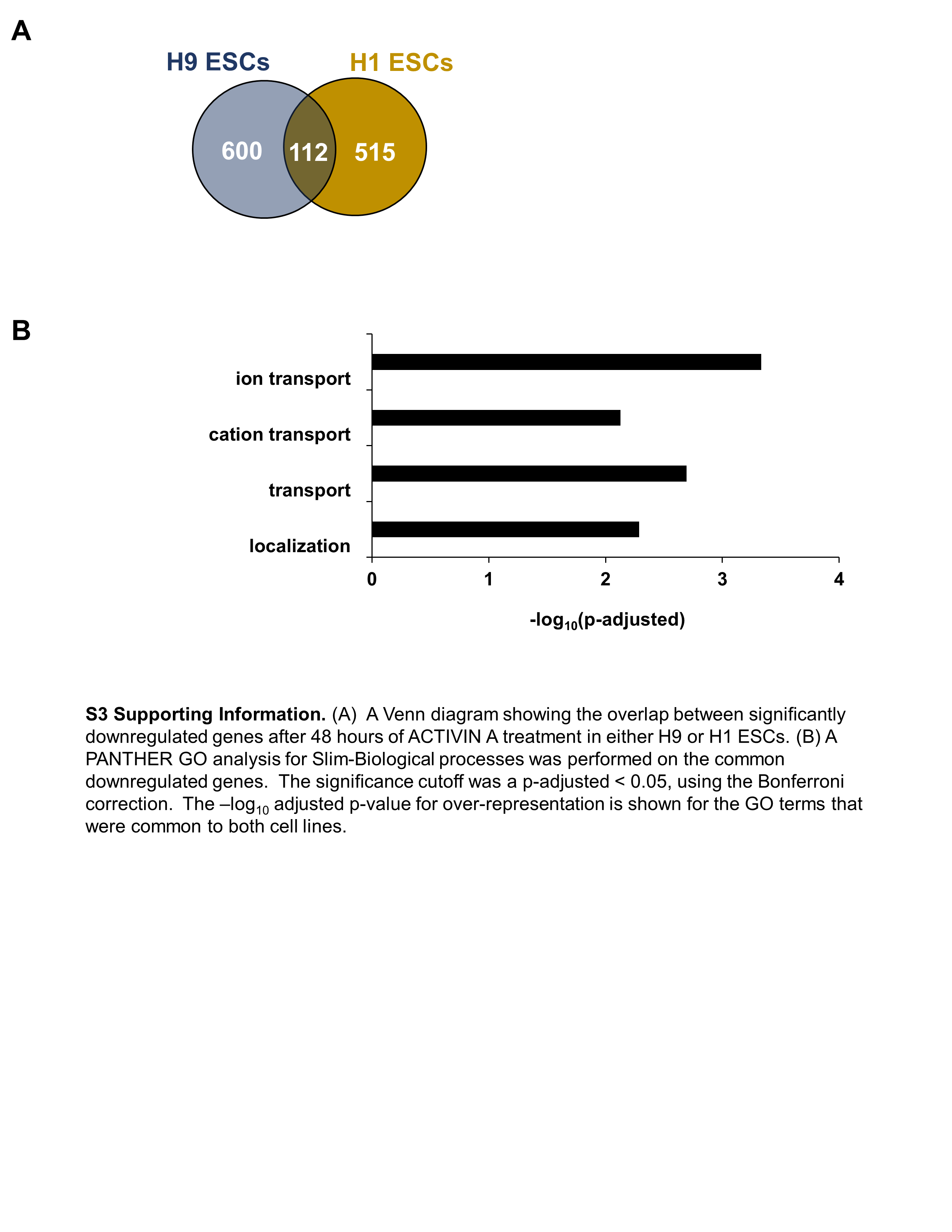

Supplement: S3 Supporting Information — (A) A Venn diagram showing the overlap between significantly downregulated genes after 48 hours of ACTIVIN A treatment in either H9 or H1 ESCs. (B) A PANTHER GO analysis for Slim-Biological processes was performed on the common downregulated genes. The significance cutoff was a p-adjusted < 0.05, using the Bonferroni correction. The–log10 p-value for over-representation is shown for the GO terms that were common to both cell lines. (TIF) [file pone.0157276.s003.TIF]

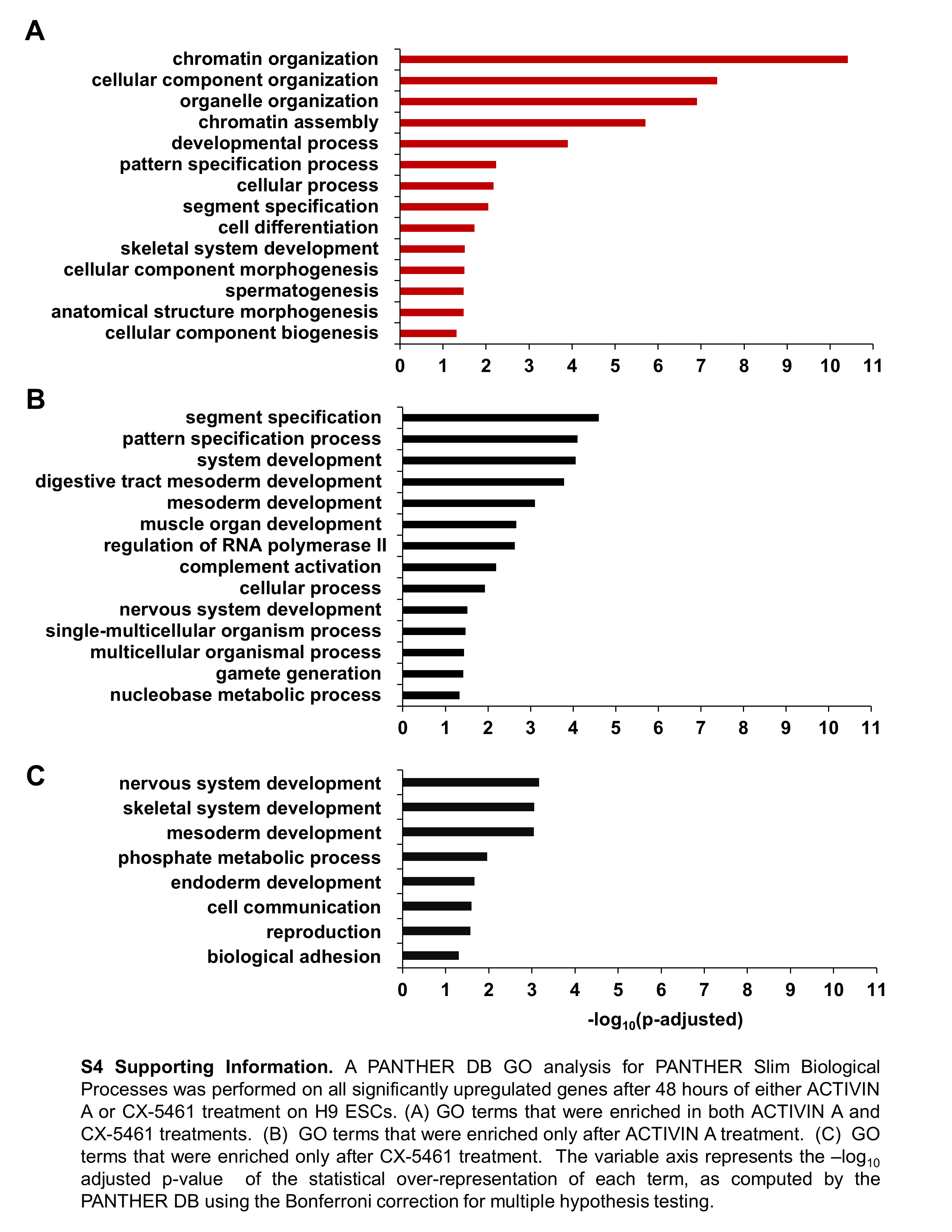

Supplement: S4 Supporting Information — (A) GO terms that were enriched in both ACTIVIN A and CX-5461 treatments. (B) GO terms that were enriched only after ACTIVIN A treatment. (C) GO terms that were enriched only after CX-5461 treatment. The variable axis represents the–log10 adjusted p-value of the statistical over-representation of each term, as computed by the PANTHER DB using the Bonferroni correction for multiple hypothesis testing. (TIF) [file pone.0157276.s004.TIF]

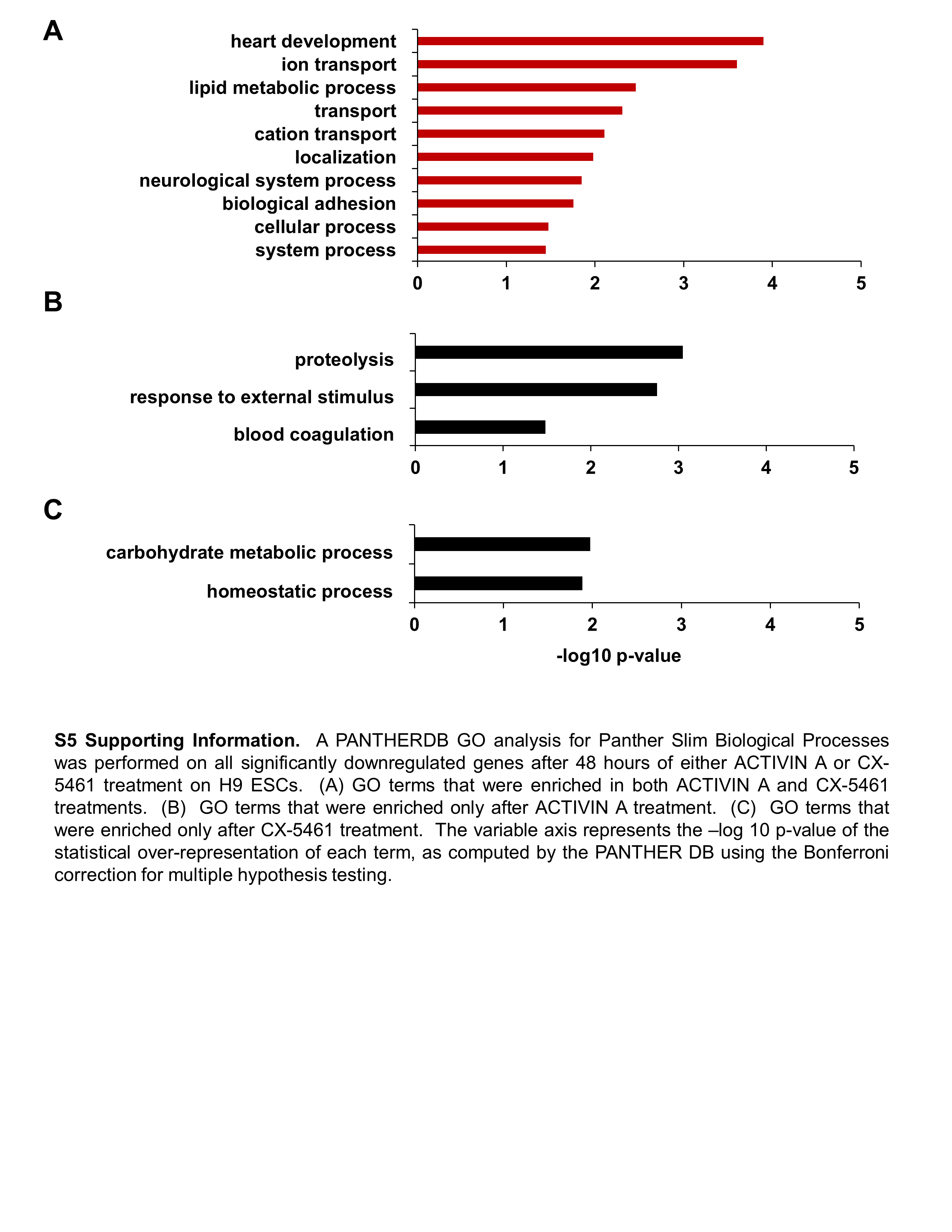

Supplement: S5 Supporting Information — (A) GO terms that were enriched in both ACTIVIN A and CX-5461 treatments. (B) GO terms that were enriched only after ACTIVIN A treatment. (C) GO terms that were enriched only after CX-5461 treatment. The variable axis represents the–log10 adjusted p-value of the statistical over-representation of each term, as computed by the PANTHER DB using the Bonferroni correction for multiple hypothesis testing. (TIF) [file pone.0157276.s005.TIF]
